# P-296. Risk Factors and Treatment Outcome of Multidrug-Resistant *Acinetobacter baumanii* Pneumonia with or without COVID-19 Infection in an Infectious Diseases Hospital from January 2020 to December 2022: A Retrospective Cohort Study

**DOI:** 10.1093/ofid/ofae631.499

**Published:** 2025-01-29

**Authors:** Ryan R Yanes, Edna M Edrada, Rontgene Solante

**Affiliations:** San Lazaro Hospital, Manila, Lingayen, Pangasinan, Philippines; SAN LAZARO HOSPITAL, MANILA, National Capital Region, Philippines; San Lazaro Hospital, Manila, National Capital Region, Philippines

## Abstract

**Background:**

*Acinetobacter baumanii* has become one of the most virulent pathogens for most hospitalized patients worldwide. Compared to the United States, Asia has a higher incidence and percentage of multidrug resistance (MDR). With the emergence of COVID-19 infection, patient's risk factors and outcomes are important to understand the correlation of the two disease entities.

**Methods:**

Single-center, retrospective cohort study that utilized records to determine the risk factors and clinical outcome of adult patients with Multidrug-resistant *Acinetobacter baumanii* pneumonia admitted at San Lazaro Hospital for a 3 year period. Cox regression analysis was employed to determine predictors for mortality.

**Results:**

A total of 70 patients were included and 9 (12.8%) of which were seen to have COVID-19. With regards to *Acinetobacter baumanii* resistance; 51.4% had Multidrug-resistance, 21.4% had Pandrug-resistance, and 27.1% had Extensively drug-resistance. In terms of treatment, most patients had Meropenem (31.4%) or Meropenem + other antibiotics (27.4%). The mean length of hospitalization did not differ significantly between non-COVID-19 (21.4 days) and COVID-19 patients (18.4 days) (p=0.711). Also, mortality rates did not differ significantly between non-COVID-19 (60.7%) and COVID-19 patients (55.6%) (p=0.771). Having Endotracheal tube was seen to be a significant predictor for mortality among patients with Multidrug-resistant *Acinetobacter baumanii pneumonia* (HR=5.0, 95%CI=1.4 to 16.4, p< 0.0001). On the other hand, Meropenem use was significantly associated with improved survival whether in single (HR=0.3, 95%CI=0.08 to 0.8, p=0.025), or in combination (HR=0.2, 95%CI=0.07 to 0.8, p=0.026).

**Conclusion:**

Among patients with Multidrug-resistant *Acinetobacter baumanii* pneumonia, length of hospital stays, and mortality rates did not differ significantly between those with and without COVID-19. Being on an Endotracheal tube increases the risk of mortality compared to those without invasive procedures. Meropenem with or without combination was seen to be effective in reducing the risk of mortality in this study.

**Disclosures:**

**All Authors**: No reported disclosures

